# How to Define the Gastroesophageal Junction

**DOI:** 10.1111/den.70193

**Published:** 2026-06-08

**Authors:** Ken‐ichi Mukaisho, Sachiko Kaida, Takahisa Nakayama, Masaji Tani, Michael Vieth, Takanori Hattori, Ryoji Kushima

**Affiliations:** ^1^ Education Center for Medicine and Nursing Shiga University of Medical Science Otsu Japan; ^2^ Department of Gastrointestinal and General Surgery Shiga University of Medical Science Otsu Japan; ^3^ Division of Human Pathology, Department of Pathology Shiga University of Medical Science Otsu Japan; ^4^ Institute of Pathology Friedrich‐Alexander‐Universität Erlangen‐Nürnberg, Klinikum Bayreuth Bayreuth Germany

**Keywords:** Barrett's epithelium, cardiac‐type mucosa, distal end of the palisade vessels, gastroesophageal junction

## Abstract

Adenocarcinomas at the gastroesophageal junction (GEJ) are increasingly common worldwide. Current classifications, such as Siewert type II and Nishi, provide a foundation, but establishing a transparent and widely accepted framework could considerably enhance identification and treatment efforts. The recent definition proposed at the Kyoto international consensus conference defines the GEJ zone as the area 1 cm above and below the distal end of the palisade vessels, marking an important step forward. Research has shown that cardiac mucosa can be present from the fetal stage, with its extent often increasing due to conditions like gastroesophageal reflux and age. This phenomenon occurs as an adaptation to the microenvironment in response to, for example, reflux stimuli, and the stem cells responsible for cardiac‐type mucosa are likely located in the gastric epithelium. However, intestinal metaplasia frequently arises from the stem cells located in the basal layer of squamous epithelium. CDX2 expression plays a significant role in the development of intestinal metaplasia. In the current classification of GEJ cancers, GEJ adenocarcinoma may represent a mix of what should be classified as esophageal adenocarcinoma (EAC) and non‐GEJ gastric cancer. The Kyoto international consensus facilitates improved differentiation among EAC, GEJ cancer, and gastric adenocarcinoma. Furthermore, we will briefly discuss endoscopic treatment, chemotherapy, and surgical treatment for GEJ cancer. Looking ahead, the reclassification of GEJ cancers using the Kyoto international consensus, coupled with comprehensive molecular analyses of tumors, promises to yield valuable insights.

The incidence of adenocarcinomas at the gastroesophageal junction (GEJ) and cardia is increasing worldwide, including in Japan. This increase is attributed to lifestyle changes, high rates of obesity, frequent occurrences of gastroesophageal reflux disease, and the declining prevalence of *Helicobacter pylori* infection [1, 2, 3, 4, 5]. Consequently, the surgical management of this condition has garnered considerable interest [6, 7, 8, 9]. However, there is currently no global consensus on the definition of GEJ, which forms the basis for the GEJ adenocarcinoma classification. According to the globally recognized classification, a Siewert type II tumor is an adenocarcinoma located between 1 and 2 cm above or below the GEJ (Figure 1A). This tumor typically originates from the epithelium of the cardiac‐type mucosa or from Barrett's epithelium at the GEJ, making it a “true carcinoma of the cardia” [8]. In contrast, the Nishi classification, widely used in Japan: any tumor located within 2 cm above or below the GEJ, regardless of its histological subtype, should be classified as GEJ cancer [10, 11, 12, 13] (Figure 1B). Nishi et al. actually proposed two types of cancer in Japanese literature: cardiac cancer extending 2 cm above and below the GEJ, and esophagogastric junction cancer extending 1 cm above and below the junction (Figure 1C). Among these, the Nishi classification, widely used in Japan, was initially proposed for cardiac cancer.

**FIGURE 1:**
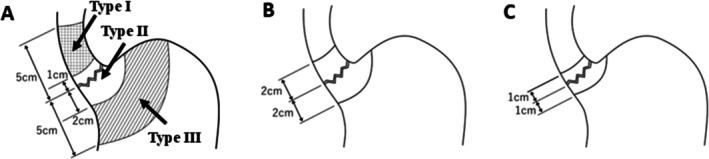
Diagram showing various classifications of GEJ cancer. (A) Siewert classification based on the tumor's epicentral location relative to the GEJ: Type I (1–5 cm above the GEJ), type II (1 cm above to 2 cm below the GEJ), and type III (2–5 cm below the GEJ). (B) The Nishi classification (Cardiac cancer): Widely used in Japan. (C) Esophagogastric junction cancer extending 1 cm above and below the junction: Nishi et al. proposed it, but it is not widely used in Japan. This area is identical to the GEJZ designated at the Kyoto International Conference.

This review aimed to clarify the concept and unique pathophysiological factors influencing the definition of the GEJ, thereby enhancing the understanding, classification, and management of diseases, particularly malignant tumors, that occur in this area.

## 1 Various Anatomical Landmarks of GEJ

**FIGURE 2:**
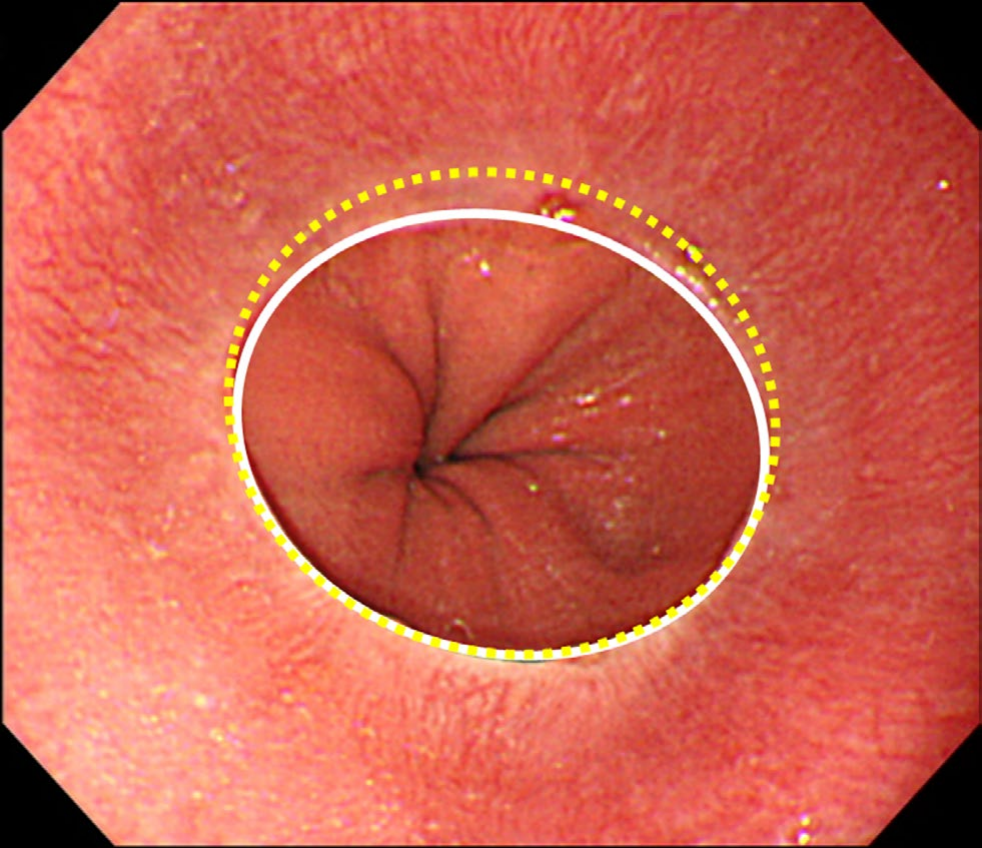
An endoscopic image displaying the SCJ, the PEGF, and the DEPV. The white line indicates the SCJ, and the yellow dashed line indicates the DEPV. In this case, the SCJ and DEPV overlap to some extent. However, the PEGF is located slightly more anally than these.

The cardia is located just below the lower end of the esophagus. Studies indicate that it is a small, poorly defined area extending a few mm up to a few cm from the GEJ and can enlarge with age [14]. However, there is neither global consensus on the definition of the GEJ nor a proper definition of cardiac‐type mucosa, leading to ongoing debates regarding the precise location of the cardia. The GEJ is characterized by several anatomical landmarks, including the squamocolumnar junction (SCJ), the proximal end of the gastric folds (PEGF), and the distal end of the palisade vessels (DEPV) [15]. Although PEGF is mainly used during endoscopic examinations, the DEPV can identify the esophageal end not only during endoscopy but also in resected specimens (Figure 2) [16, 17, 18]. The DEPV marks the anatomical termination of the lower esophageal sphincter and the transition into the gastric muscle structures [19, 20]. This landmark is also used as a reference point for identifying the lower esophageal sphincter during peroral esophageal myotomy, a procedure used to treat achalasia [21, 22]. Importantly, this vascular landmark remains unaffected by esophageal columnar metaplasia or gastric pathologies, such as atrophy and intestinal metaplasia. A recent review indicated that the DEPV is a more accurate mucosal landmark for the GEJ than the PEGF [23]. In Western populations, the DEPV is believed to be absent or less visible; however, this does not appear to hold true when adhering to the precise definition of the landmark. While the DEPV is often regarded as the most anatomically accurate landmark for the GEJ in Japan, a global consensus on this matter has yet to be reached.

## 2 Novel Concepts Regarding the Gastroesophageal Junction Zone (GEJZ)

In March 2019, the Kyoto International Conference was held to discuss various issues related to the GEJZ and present new concepts and challenges to the global scientific community [24]. The conference concluded that the GEJ should be defined endoscopically using the DEPV as reference. Additionally, owing to the presence of cardiac glands near the GEJ, the region extending 1 cm proximally and 1 cm distally to the DEPV was designated the GEJZ [24] (Figure 1c). At the Kyoto International Conference, cancers arising in the GEJZ, as defined above, were proposed as a substitute for Siewert type II cancer in the junctional zone [24]. Recently, Ota et al. validated this definition of GEJZ by immunohistochemical staining for MUC6 (a marker of pyloric glands‐type mucin), H+/K + ‐ATPase (a marker of parietal cells), and pepsinogen I (a marker of chief cells) in mucosal specimens from the GEJ that had been resected through endoscopic submucosal dissection to treat refractory gastroesophageal reflux disease [25]. The study clearly shows that GEJZ can be defined as the area 1 cm above and below the GEJ, based on the distribution of cardiac glands. This finding provides a strong histological basis for classifying the GEJZ, thereby enhancing the accuracy of GEJ cancer classification and improving diagnostic consistency in clinical and research settings.

## 3 Existence and Histology of Cardiac‐Type Mucosa at the GEJ at Birth

**FIGURE 3:**
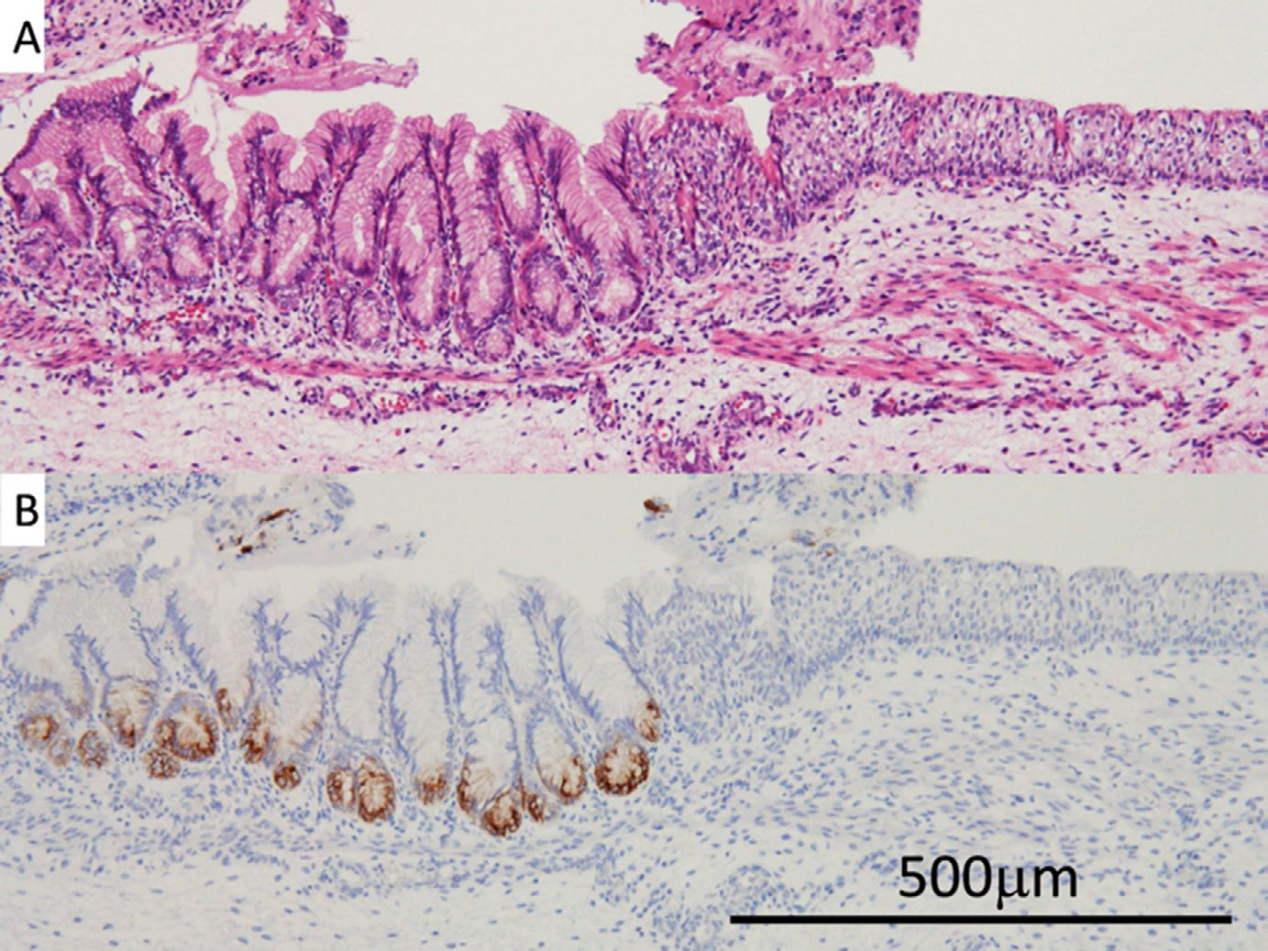
Cardiac mucosa in fetus. (A) HE stains, (B) immunohistochemical staining for MUC6. Cardiac mucosa was observed during a postmortem examination of a fetus. In Figure 1A, the cardiac glands appears unclear in the HE stains, but in the MUC6 immunostaining, MUC6‐positive cardiac glands can be confirmed in the deeper part of the columnar epithelium. In MUC6 immunostaining, a positive result is indicated by a brown color, which is achieved through the use of 3,3′‐diaminobenzidine(DAB)for visualization.

Some literature refers to “cardiac mucosa” and “cardiac‐type mucosa” as synonymous terms. However, the use of “cardiac‐type mucosa” often suggests that the author is indicating the presence of acquired components in the mucosa. Several studies have examined fetuses or newborns who died shortly after birth and concluded that these findings are part of normal embryonic gastric development [26, 27, 28, 29, 30] (Figure 3). In contrast, Chandrasoma et al. proposed that the cardiac glands develop as a metaplastic response of the esophageal squamous epithelium due to reflux injury. They asserted that native cardiac glands do not exist; rather, all cardiac glands are acquired [31, 32, 33, 34, 35]. Therefore, the existence and histology of cardiac mucosa at the GEJ at birth remain controversial. However, Takubo et al. recently reported that cardiac mucosa does exist in neonates and infants, defining it as oxyntocardiac mucosa, regardless of the presence or absence of parietal cells [36]. For the clinical question of whether cardiac mucosa exists in fetuses and infants, evidence indicates that it does occur, although the extent is minimal and enlarges with age in the Kyoto international consensus conference. The agreement percentages were as follows: 82% strongly agreed, 18% agreed with minor reservations, 0% disagreed with major reservations, and 0% strongly disagreed [24]. Therefore, it is consensual that cardiac‐type mucosa is present in the fetal stage and may increase physiologically and due to conditions, such as gastroesophageal reflux disease.

## 4 Histological Features of the Cardiac‐Type Mucosa

The gastric mucosa exhibits a consistent histological pattern throughout its length. It consists of a superficial layer of foveolae, which are invaginations of the surface epithelium, and a deeper layer composed of coiled glands that open at the base of the foveolae [14]. The cardiac‐type mucosa, located adjacent to the GEJ, contains glands that secrete mucus [14]. The pyloric mucosa extends proximally from the pylorus and includes the mucous‐secreting glands. The cells within these glands are morphologically similar, thus complicating the distinction between specimens from the pyloric and cardiac mucosa when their specific locations are unknown. Both mucous and mixed glands, which contain mucous and parietal cells (known as oxyntocardiac glands), are components of cardiac‐type mucosa [24, 36]. In strict histological terms, glands lacking parietal cells are classified as cardiac glands, whereas those containing parietal cells are identified as oxyntocardiac glands. In contrast, the mucosa in other areas of the stomach, such as the corpus and fundus, specializes in secreting acid and pepsin. Several studies conducted on adults have identified areas of cardiac‐type mucosa within the GEJ [37, 38, 39]. However, these studies did not differentiate between pyloric/pseudopyloric metaplasia and cardiac or oxyntocardiac glands because of their reliance on hematoxylin–eosin (H&E) staining, which lacks the ability to provide immunohistochemical differentiation. The immunostaining results for the cardiac glands, oxyntocardiac glands, oxyntic mucosa, pseudopyloric metaplasia, and pyloric metaplasia are summarized in Table 1. Ota et al. suggested that the mucosa at the cardiac–oxyntic border may undergo metaplasia into pyloric or pseudopyloric types, leading to the expansion of the cardiac‐type mucosa toward the distal side. The cardiac‐type mucosa varies in size depending on various inflammatory conditions and includes the following elements: native cardiac mucosa located at the GEJ [25]; columnar metaplastic mucosa, which occurs in the lower esophagus due to reflux esophagitis; and pyloric or pseudopyloric metaplasia, which arises in the oxyntic region near the GEJ in response to gastric atrophy associated with chronic inflammation [40, 41, 42].

**TABLE 1:** The summary of immunostaining results for various lesion at the GEJ.

	Pyloric gland‐type mucin	Parietal cells	Chief cells
	(MUC6)	(H^+^/K^+^‐ATPase)	(Pepsinogen I)
Cardiac gland	Positive	Negative	Negative
Oxyntocardiac gland	Positive	Positive	Negative
Oxyntic mucosa	Positive	Positive	Positive
Pseudopyloric metaplasia	Positive	Negative/positive	Positive
Pyloric metaplasia	Positive	Negative	Negative

## 5 Definition of Barrett's Esophagus (BE)

**FIGURE 4:**
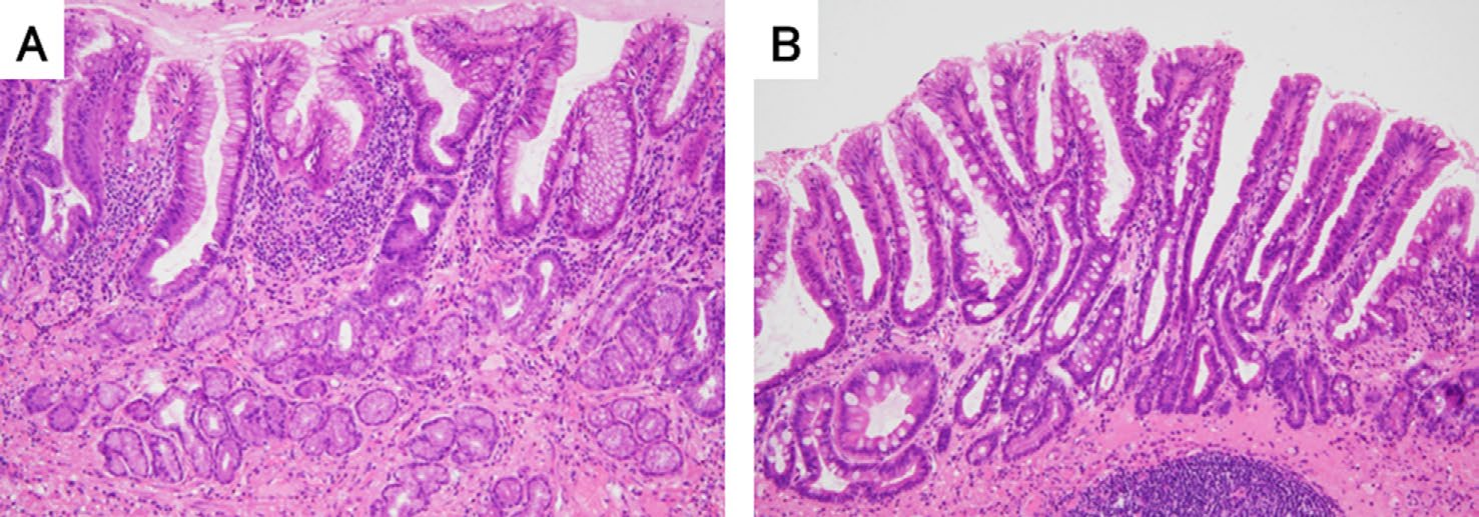
Barett's epithelium (human sample). (A) Cardiac‐type mucosa, (B) intestinal metaplasia. Intestinal goblet cells cannot be identified in Figure 4A. In Figure 4B, goblet cells are prominently observed, but there is also a mixture of gastric‐type epithelium and intestinal metaplasia present within the BE.

International guidelines for diagnosing Barrett's esophagus (BE) differ notably in two aspects: the required length of metaplastic mucosa and the necessity for intestinal metaplasia—also known as specialized columnar epithelium or specialized intestinal metaplasia [43, 44, 45, 46, 47, 48] (Figure 4). Because the Japan Esophageal Society accepts any length of metaplastic change, referring to BE segments less than 1 cm as ultrashort‐segment BE (USSBE), the diagnosis of USSBE is much more frequent in Japan than elsewhere [49]. In contrast, the most recent AGA guideline no longer defines segments < 1 cm as BE and instead uses the term “columnar‐lined esophagus < 1 cm with intestinal metaplasia” [50]. Other societies also require a minimum length of 1 cm for the diagnosis of BE [51, 52]. Thus, Japan is currently the only major society that still classifies segments < 1 cm as BE.

Although USSBE has a negligible malignancy risk [53, 54, 55, 56, 57, 58, 59, 60], it often triggers disproportionate cancer anxiety, which is further exacerbated by international diagnostic differences—particularly the Western requirement for intestinal metaplasia. Furthermore, previous studies have consistently reported that interobserver and interinstitutional agreement for the diagnosis of USSBE is very poor, while that for non‐USSBE is at least moderate [53]. As a result, inconsistent and unstable diagnoses are frequent for USSBE, making it difficult for affected patients to develop an appropriate understanding of BE carcinogenesis; in some cases, they may even ignore the diagnosis entirely. This may explain the scattered distribution of the cancer worry scale, which was originally developed to quantitatively assess concerns about cancer recurrence and the impact of these concerns on daily functioning in patients who had undergone treatment for cancers, in USSBE patients observed in the literature [49]. The diagnostic significance of USSBE may therefore require reevaluation. Endoscopists should balance rigorous diagnosis with clear communication and consider adopting higher diagnostic thresholds (e.g., 1 cm) to reduce unnecessary patient anxiety.

Additionally, the GEJ, which is a landmark used to assess the length of BE, as recommended by most guidelines, is imprecise. For instance, its location can vary with respiration and the extent of insufflation, rendering measurements of the metaplastic mucosa inaccurate and unreliable. Furthermore, a study involving healthy volunteers reported that expansion of the cardia occurs through columnar metaplasia of the distal esophagus and is worsened by central obesity. This metaplastic origin of the expanded cardia may explain the substantial number of cardia adenocarcinomas that cannot be attributed to *H. pylori* or transsphincteric acid reflux [61]. Thus, determining a global definition of BE may be difficult.

## 6 Origin of Stem Cells in Cardiac‐Type Mucosa of the Esophagus

**FIGURE 5:**
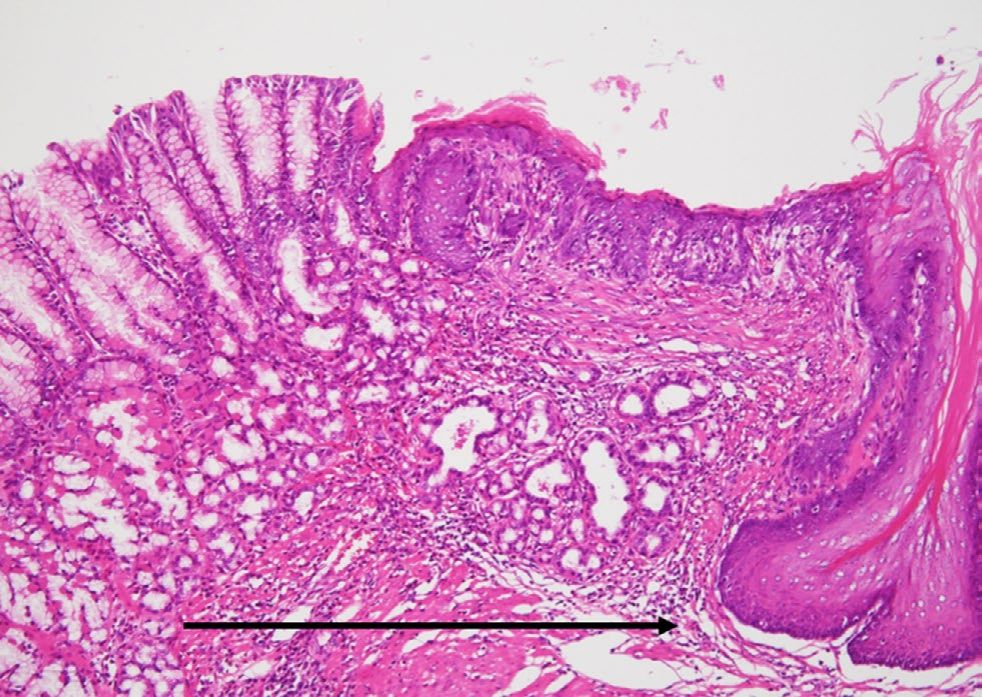
Cardiac‐type mucosa (rat reflux model). The figure illustrates a tissue specimen from the esophagogastric junction in a rat reflux model, which mimics gastric reflux containing duodenal fluid, similar to that seen in humans. The right side of the specimen represents the oral side, whereas the left side represents the caudal side. The surface layer displays regenerative stratified squamous epithelium. On the oral side, there is evidence of hyperplastic stratified squamous epithelium. In instances of erosions or ulcers in the esophagus, the cardiac‐type mucosa extends toward the oral side to facilitate the repair of these lesions (black arrow). Additionally, on the left side of the figure, atrophy occurs alongside the development of pseudopyloric metaplasia, leading to the appearance of the cardiac‐type mucosa extending toward both the oral and caudal sides.

**FIGURE 6:**
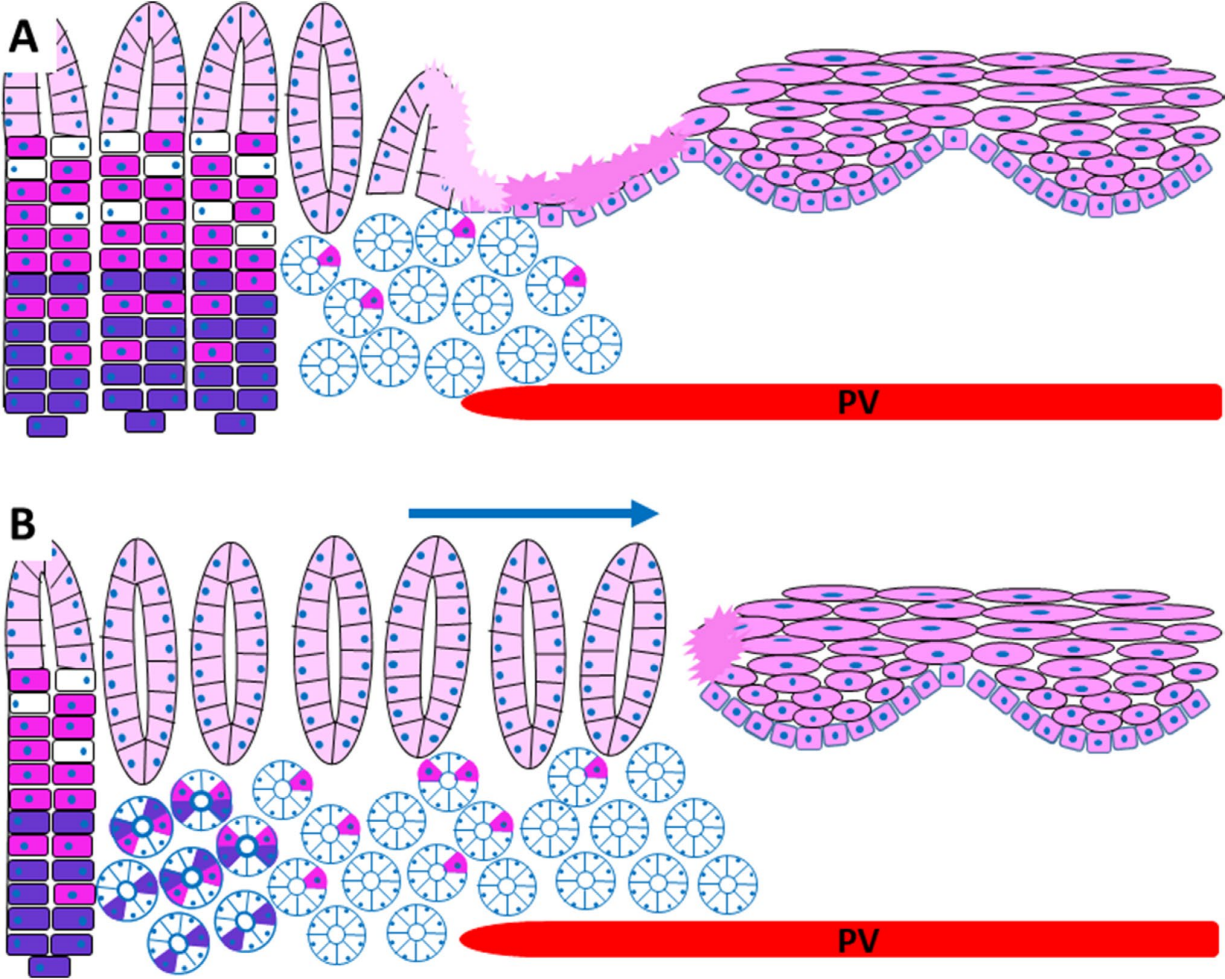
Schematic illustration of the formation process of columnar‐line esophagus at the GEJ. (A) Erosion at the GEJ, (B) repairing the GEJ erosion with cardiac‐type mucosa. PV: Palisade vessel. The esophagus is covered by stratified squamous epithelium. On the left side, there are oxyntic glands. The cells with pink cytoplasm are parietal cells, whereas those with purple cytoplasm represent the chief cells. The cells with white cytoplasm in the oxyntic mucosa on the left are mucous neck cells. The glands outlined with light blue lines are the cardiac glands, and those containing parietal cells are referred to as oxyntocardiac glands. The mucosa covered by foveolar epithelium has cardiac glands with occasional chief or parietal cells, indicating pyloric or pseudopyloric metaplasia. The blue arrow shows that the cardiac‐type mucosa extends distally to heal ulcers and erosions in the esophagus.

**FIGURE 7:**
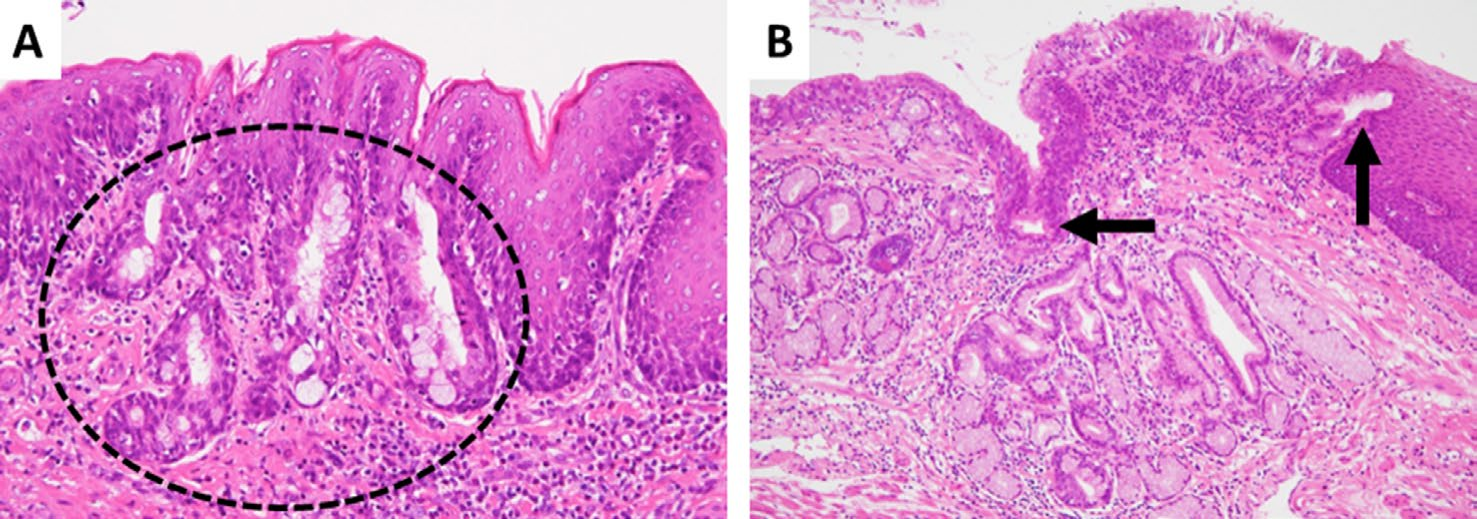
Gastric metaplasia observed in a location distant from the GEJ toward the oral side. (A) Rat duodenal contents reflux model, (B) human case. In the reflux model, the columnar epithelium develops within the regenerative stratified squamous epithelium (black dashed line) (A). In human cases, duct‐like structures (black arrows) emerge from the stratified squamous epithelium, or pyloric gland metaplasia occurs from the cells in the ducts of the esophageal glands (B).

When an ulcer develops in the stratified squamous epithelium of the esophagus near the upper margin of the stomach, the cardiac‐type mucosa probably extends toward the oral side [40, 41]. When reflux is present and erosions or ulcers occur, the stem cells of the esophageal stratified squamous epithelium are less likely to differentiate into stratified squamous epithelium. This adaptation is a response to the changing microenvironment. As a result, when reflux is ongoing, cardiac‐type mucosa can extend from the gastric side toward the oral side. In cases where the epithelial defect is extensive, it is often replaced by foveolar epithelium, which may be accompanied by intestinal metaplasia and typically lacks cardiac glands. The original cells (stem cells) of the cardia glands or glandular foveolar epithelium that migrate orally are derived from the gastric epithelium and not from the esophageal stratified squamous epithelium. This phenomenon does not represent a transformation of the cells of the stratified squamous epithelium of the esophagus into columnar epithelium (Figures 5 and 6). In contrast, pyloric‐type glands, which are known as pyloric or pseudopyloric metaplasia and are positive for MUC6 (similar to cardiac‐type glands), can occur in various parts of the body as a result of metaplastic changes [62, 63, 64, 65, 66, 67, 68, 69, 70]. In the rat duodenal reflux model and human tissue, pyloric gland metaplasia occurs in the esophagus away from the GEJ (Figure 7A,B).

## 7 What Cells Are Responsible for Intestinal Metaplasia?

### 7.1 Cells That Have the Potential to Develop Into Intestinal Metaplasia

Intestinal metaplasia was more frequently observed in LSBE than in USBE or SSBE. Cardiac‐type mucosa is thought to originate from native cardiac mucosa; however, there is an ongoing debate regarding the source of intestinal metaplasia, particularly in LSBE. Several theories have been proposed on this topic, including (i) the columnar epithelium may develop directly from the esophageal squamous epithelium [71, 72, 73, 74, 75], (ii) it may originate from gastric mucosa [40, 41, 76], (iii) the esophageal gland ducts may be involved [77], (iv) the mucosa at the esophagogastric junction may play a role [78], (v) it might derive from fetal remnants [79], (vi) bone marrow cells could contribute [80], and (vii) it may be a result of wound repair [81]. Our research using rats lacking esophageal glands suggests that intestinal metaplasia likely originates from stem cells located in the basal layer of the esophageal stratified squamous epithelium [73, 74]. In humans, stem cells located in the basal layer of the stratified squamous epithelium of the esophagus or in the ducts of the esophageal glands, particularly those situated distally from the GEJ, are likely the origins of intestinal metaplasia.

### 7.2 Role of CDX2 Expression in the Development of Intestinal Metaplasia

**FIGURE 8:**
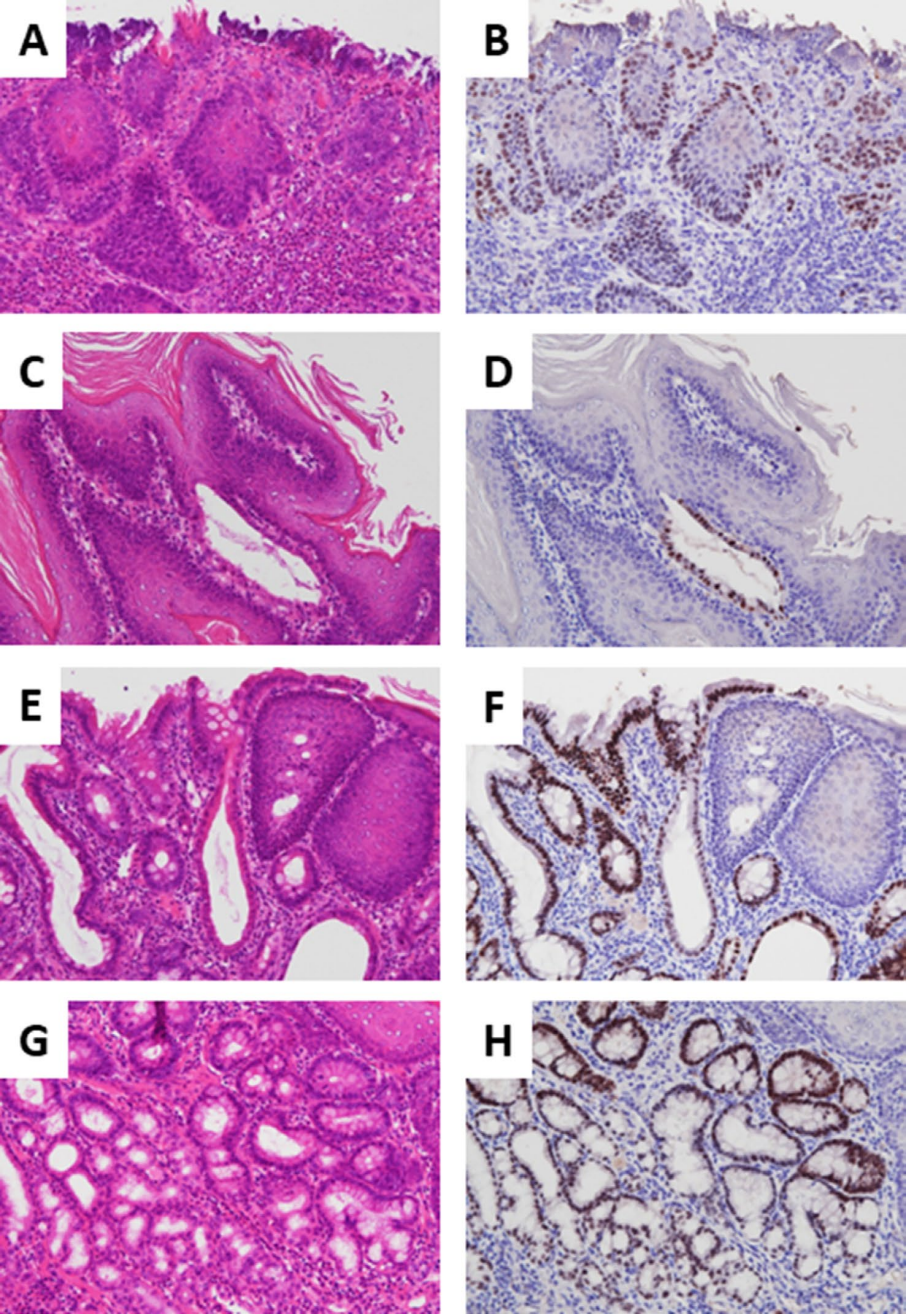
CDX2 expression in the development of intestinal metaplasia. (A, C, E, G) HE stains. (B, D, F, H) Immunohistochemical staining for CDX2. In CDX2 immunostaining, a positive result is indicated by a brown color, which is achieved with DAB for visualization. CDX2 expression is crucial for the development of Barrett's epithelium with goblet cells.

**FIGURE 9:**
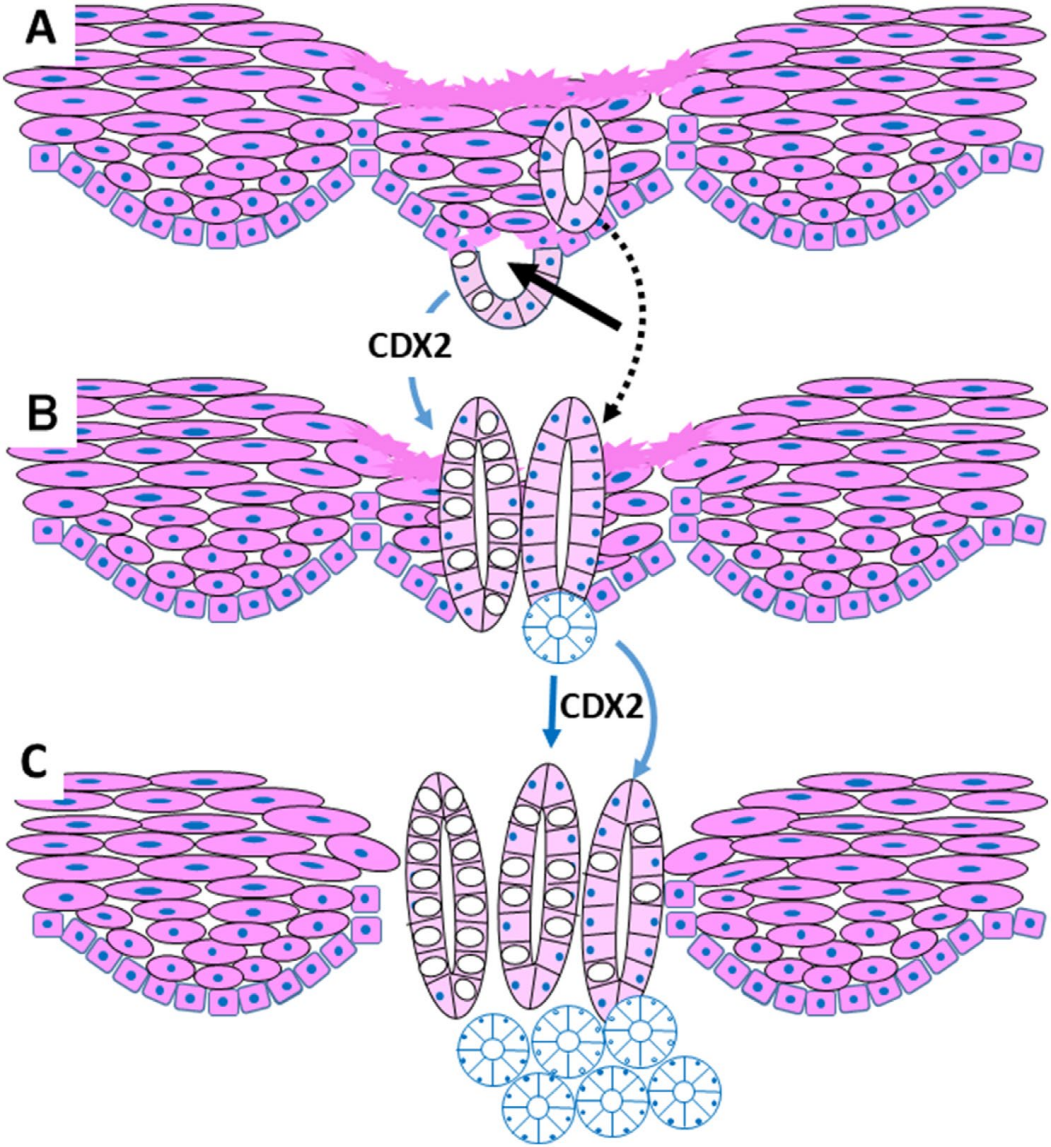
Schematic illustration of the formation process of Barrett's epithelium at a location distant from the GEJ. The early stages of the lesion include the formation of gastric metaplasia and intestinal metaplasia. The white oval shapes in the diagram represent intestinal goblet cells. Due to the effects of reflux, columnar epithelium develops within the stratified squamous epithelium (as shown in A), leading to the elongation of epithelial projections. Over time, gastric metaplasia occurs (B), which is followed by the development of intestinal metaplasia resulting from pyloric gland metaplasia (9C). CDX2 expression is critical in this process. When reflux acts as a strong stimulus, CDX2 is expressed directly in the stratified squamous epithelium, promoting the formation of duct‐like structures that arise from this epithelium. Ultimately, this process results in intestinal metaplasia, which is characterized by the presence of goblet cells.

**FIGURE 10:**
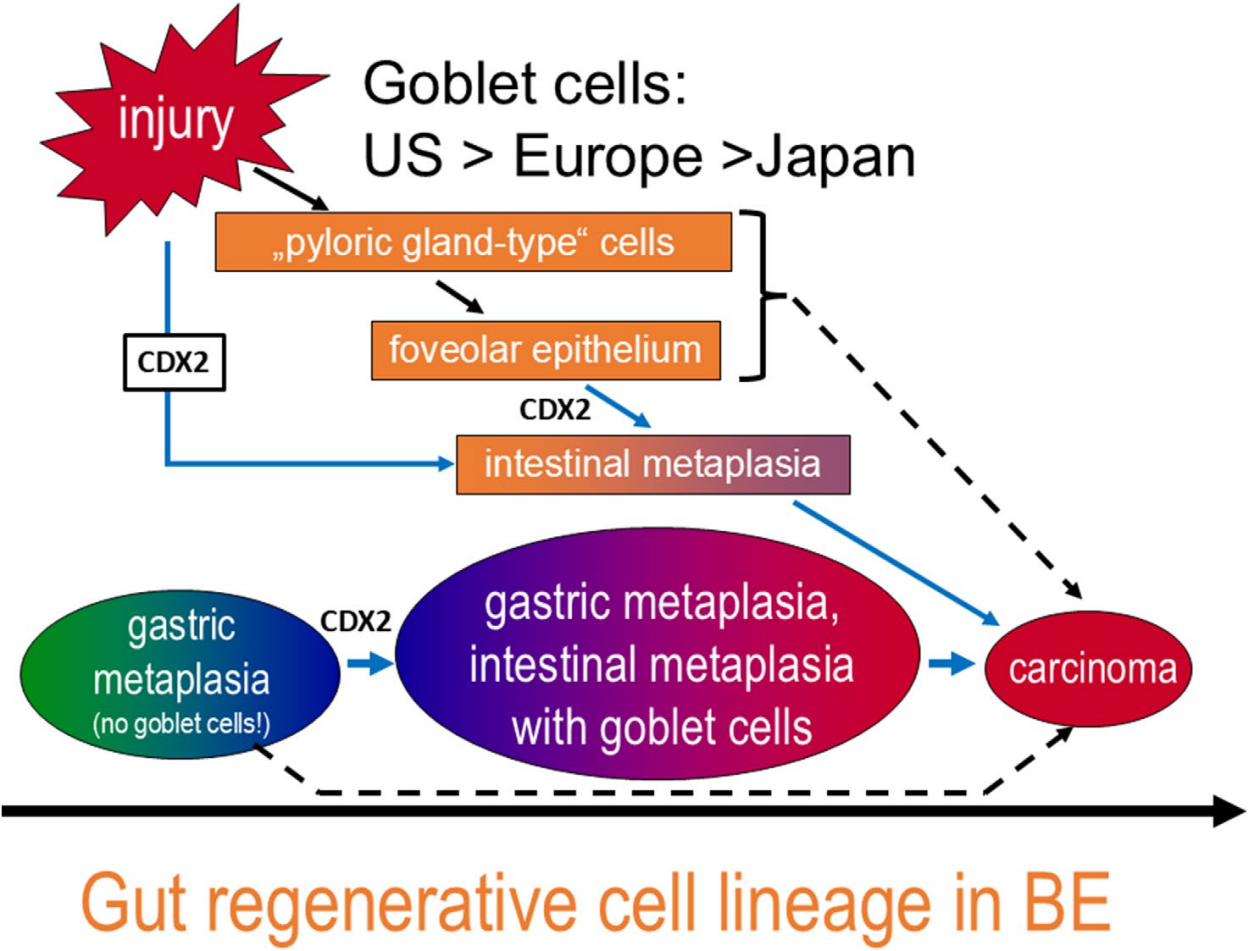
Occurrence of intestinal metaplasia in the GRCL process. This figure illustrates the concept of the GRCL (gut regenerative cell lineage) carcinogenesis. In response to mucosal breaks, the gut undergoes a regeneration process where pyloric gland cells (deep gastric glands) first appear, followed by foveolar cells (gastric surface epithelium), leading to the replacement of the layered squamous epithelium by gastric epithelium including stromal tissue (gastric metaplasia). Subsequently, intestinal‐type goblet cells may emerge and become intermingled with foveolar cells. This progression is known as GRCL. The blue line indicates the transition to the intestinal type cells. CDX2 expression is important at this stage. Depending on the degree of stimulation, either gastrointestinal mixed type metaplasia occurs via gastric metaplasia or intestinal metaplasia occurs directly. EACs arising from BE are believed to develop from this lineage. Intestinal metaplasia with goblet cells occurs more frequently in Western populations than in Japanese individuals.

A previous study indicated that CDX2 is expressed in the stratified squamous epithelium of a rat reflux model [82]. Additionally, another research effort using a mouse model revealed a novel finding: the overexpression of CDX2 in the absence of P63 in the mouse esophageal epithelium led to the development of Barrett's‐like metaplasia in vivo [83]. This condition is characterized by the presence of columnar epithelium with an intestinal crypt‐like structure that expresses intestinal markers. In our duodenal fluid reflux models, we found that CDX2 is expressed during the early stages of intestinal metaplasia (Figure 8). Figure 9 illustrates the process of intestinal metaplasia. In the reflux models, CDX2 is expressed in the basal layer of the stratified squamous epithelium, which experiences repeated erosion and regeneration (Figure 8A,B). This leads to the development of CDX2‐expressing columnar epithelium from the stratified squamous epithelium in a duct‐like manner, ultimately forming glandular lumens (Figure 8C,D). Subsequently, intestinal‐type epithelium containing goblet cells emerges (Figure 8E,F). Throughout this process, pyloric gland metaplasia also occurs, although CDX2 is only weakly expressed in these pyloric glands (Figure 8G,H). Thus, CDX2 is strongly expressed in intestinal‐type cells with goblet cells, whereas it is weakly expressed in gastrointestinal mixed‐type cells. It has been suggested that pyloric gland‐type cells develop from these stem cells during the process of intestinal metaplasia (Figures 9 and 10) [40, 74].

## 8 Developmental Process of GEJ Cancer

**FIGURE 11:**
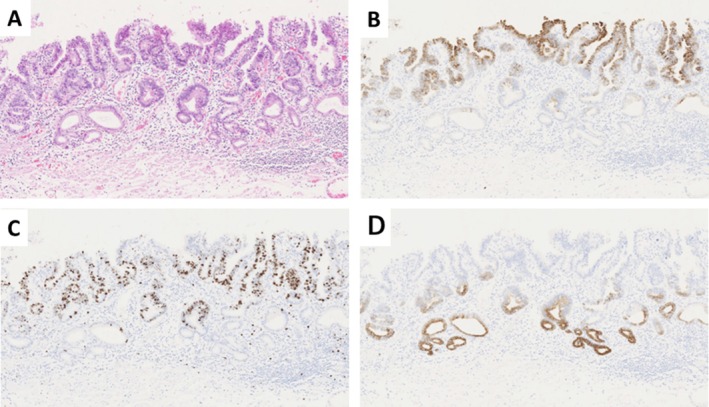
Early lesion of gastric‐type GEJ adenocarcinoma. (A) HE staining, (B) Immunohistochemical staining for MUC5AC, (C) Ki67, (D) MUC6. In immunostaining, a positive result is indicated by a brown color, which is achieved through the use of DAB for visualization. This is a GEJ adenocarcinoma that originates from cardiac‐type mucosa. The differentiation process involves the formation of glandular epithelium that is positive for MUC5AC, extending toward the lumen, while MUC6‐positive pyloric gland‐type cells develop in the deeper layers of the mucosa. A proliferative zone containing Ki67‐positive cells was observed between these two differentiated regions.

Based on our research on non‐GEJ gastric cancer, the developmental processes of gastric‐type and intestinal epithelial type tumors differ [84]. Gastric‐type tumors originate from a proliferative zone located between the glandular epithelium and the proper gastric layer [84]. In esophageal adenocarcinoma (EAC), we hypothesized that the carcinogenic processes differ between cancers that arise from cardiac‐type mucosa and those that develop from specialized intestinal‐type Barrett's epithelium [40]. In early‐stage cancers originating from the cardiac‐type mucosa, differentiation occurs in a manner similar to that of the normal gastric mucosa. This differentiation involves the development of MUC5AC‐positive glandular epithelium toward the lumen and the differentiation of MUC6‐positive pyloric gland‐type mucosa toward the deeper layers of the mucosa. A proliferative zone consisting of Ki67‐positive cells was observed between the two differentiated regions (Figure 11).

## 9 Two Distinct Etiologies of GEJ Cancer

It is widely accepted that chronic mucosal inflammation, caused by *H. pylori* infection, is carcinogen class I for gastric cancers [85]. However, the role of *H. pylori* infection in the development of cardia cancer remains unclear [86, 87, 88, 89, 90, 91]. Several studies have indicated two distinct etiologies of cardia cancer: one that is more commonly associated with GER in *H. pylori* ‐negative than in *H. pylori* ‐positive patients and resembles EAC, and another that is associated with *H. pylori* ‐induced atrophic gastritis and resembles non‐cardia cancer [92, 93, 94, 95]. Even in a molecular classification model that utilizes a Bayesian compound covariate predictor based on differential mRNA expression, a study of EAC and non‐GEJ cancer from the Cancer Genome Atlas cohort revealed that GEJ cancer comprises both EAC‐like and non‐GEJ cancer‐like groups. These groups consistently demonstrated distinct molecular characteristics [96].

## 10 Molecular Target of GEJ Cancer

The Cancer Genome Atlas revealed that most GEJ and EAC arising from BE are classified into the chromosomal instability subtype, whereas other subtypes (Epstein–Barr virus‐positive, microsatellite instability, and genomically stable) are relatively rare compared to non‐GEJ gastric adenocarcinomas [97, 98]. Most GEJs and EAC are eligible for molecular‐targeted therapy [99]. HER2 and PD‐L1 are key treatment targets for gastroesophageal adenocarcinomas. The ToGA trial found that 32.2% of GEJ adenocarcinomas tested positive for HER2 compared to 21.4% of non‐GEJ gastric adenocarcinomas [100]. In the KEYNOTE‐061 trial, 73.0% of GEJ cases had a Combined Positive Score (CPS) of ≥ 1 compared to 63.9% of non‐GEJ cases [101]. The CheckMate‐649 trial found 65.4% of GEJ cases had a CPS of five or more, along with 55.9% of EACs and 60.1% of non‐GEJ gastric adenocarcinomas [102]. A Japanese study showed that 55.0% of GEJ cases and 62.3% of non‐GEJ cases had a CPS of one or higher [103]. CLDN18 is detected in 33.3% of GEJ adenocarcinomas and 23.3% of EACs [99].

## 11 Endoscopic Treatment for GEJ Adenocarcinoma

ESD is weakly recommended as a curative option for esophageal and GEJ cancers if the following criteria are met: intramucosal carcinoma or submucosal (SM) invasion depth ≤ 500 μm, tumor diameter ≤ 3 cm, negative resection margins, absence of lymphovascular invasion, and no poorly differentiated components. This recommendation achieved 80% agreement; however, the level of evidence remains at Grade C [104]. In Japan, the criteria for additional surgical intervention after ESD differ significantly between esophageal and gastric cancers. Additional treatment is indicated for SM invasion ≥ 200 μm in esophageal cancer, whereas the threshold for gastric cancer is ≥ 500 μm [105, 106]. Currently, GEJ cancers are primarily managed according to gastric cancer guidelines; however, the clinical validity of this approach warrants further investigation. From a histological perspective, the risk of lymph node metastasis (LNM) varies between SCC and EAC. Leers et al. reported that the risk of LNM in EAC is exceedingly low (0%) when SM invasion is ≤ 500 μm, provided there is no lymphovascular invasion, and the tumor is well‐to‐moderately differentiated (G1/2) [107]. Although endoscopic therapy may be adequate in selected patients with ‘low‐risk’ sm1 EAC, submucosal SCC necessitates esophageal resection and systematic lymphadenectomy because of its aggressive nature and tendency for early metastasis [108]. Conversely, early adenocarcinomas and SCCs do not differ in their rate of lymphatic involvement. The rate of lymph‐node metastasis increases with the depth of submucosal infiltration, but metastases can already occur in sm1 lesions. Submucosal infiltration is a contraindication for endoscopic mucosectomy [109]. Given these conflicting findings, further data accumulation and clinicopathological analyses are essential to refine the indications for ESD in GEJ cancer.

## 12 Chemotherapy for GEJ Adenocarcinoma

We referenced the Clinical Practice Guidelines for GEJ Cancer established at the Upper GI Oncology Summit held in 2023 [104]. In response to whether chemotherapy for gastric adenocarcinoma, EAC, and GEJ cancer is recommended, the findings of the subgroup analyses indicated that the outcomes of patients with GEJ adenocarcinoma or EAC were similar to those of patients with gastric adenocarcinoma. Therefore, the recommendation is as follows: It is weakly recommended to use the same chemotherapy regimens established for patients with unresectable, advanced, or recurrent gastric adenocarcinoma in patients with esophagogastric junction adenocarcinoma and esophageal adenocarcinoma. This similarity is likely because, according to analyses based on previous classifications, GEJ cancer is considered a combination of EAC and gastric cancer. Retrospective studies also examined the treatment outcomes of first‐line therapies in patients with gastric adenocarcinoma and GEJ adenocarcinoma. No significant difference in overall survival was observed between the two groups [110, 111].

## 13 Surgery for GEJ Adenocarcinoma

### 13.1 Transthoracic Techniques

Surgery for GEJ adenocarcinoma varies depending on the surgeon performing the procedure, as there is no consensus on the resection technique or lymph node dissection range. Thoracic surgeons tend to perform procedures similar to those for esophageal cancer, while gastrointestinal surgeons tend to perform procedures similar to those for gastric cancer. In Japan, guidelines recommend a right thoracotomy approach for esophageal invasion lengths of 4 cm or more, and an open laparotomy or transhiatal approach if safe resection and reconstruction are technically feasible for invasion lengths of less than 4 cm. The most common transthoracic techniques are the Ivor Lewis [112] and McKeown esophagectomies [113]. The Ivor Lewis procedure combines laparotomy and right thoracotomy with an intrathoracic anastomosis above the azygos vein, making it suitable for distal thoracic lesions but less optimal for tumors in the mid‐esophagus owing to the limited proximal margin. Gastric conduit creation involves lymphadenectomy of celiac and left gastric nodes, division of the left gastric artery, and preservation of right gastric and gastroepiploic vessels. The McKeown technique is similar in abdominal and thoracic phases but includes an additional cervical incision and anastomosis, extending its applicability to tumors of the upper, middle, and lower thoracic esophagus.

### 13.2 Transhiatal Esophagectomy

Transhiatal esophagectomy performed through abdominal and cervical incisions without thoracotomy allows resection for tumors at any thoracic level [114]. However, it is technically challenging and potentially hazardous for bulky esophageal tumors near the trachea. Although some studies show lower morbidity and early survival advantages compared with transthoracic approaches [115], the reduced lymph node yield raises concerns regarding long‐term oncologic adequacy [116].

The left transthoracic or thoracoabdominal approach employs combined abdominal and left thoracic incisions, typically for bulky distal lesions, with an intrathoracic anastomosis positioned just above the inferior pulmonary vein [117].

### 13.3 Minimally Invasive Esophagectomy

Minimally invasive esophagectomy (MIE) encompassing laparoscopic‐thoracoscopic Ivor Lewis or McKeown methods has been associated with lower postoperative mortality, shorter hospital stays, and fewer pulmonary complications, with equivalent or better long‐term outcomes in certain studies [118, 119]. Hybrid MIE strategies—laparoscopic abdominal phase combined with open thoracotomy—can similarly reduce morbidity without altering survival rates [120]. However, MIE may be less suitable in cases of prior abdominal surgery, large tumors, poor gastric conduit viability, or difficult lymphadenectomy.

### 13.4 Robotic‐Assisted MIE

Robotic‐assisted MIE enhances visualization and precision in confined operative fields, offering reduced postoperative pain and respiratory complications compared with open surgery [121]. Despite its longer operative time and higher costs, oncologic outcomes appear comparable at intermediate follow‐up although further large‐scale randomized trials are needed.

## 14 Conclusion

The DEPV is widely regarded as the most precise landmark for the GEJ in Japan, yet a global consensus on its significance remains unformed. In Western populations, the DEPV may be less visible, but its importance endures. The Kyoto international consensus offers a more refined understanding of the GEJ compared to the Siewert or Nishi classifications, enabling more apparent differentiation among esophageal cancer, GEJ cancer and gastric adenocarcinoma. By reclassifying GEJ cancers through the Kyoto lens and conducting molecular analyses, we anticipate valuable insights that could transform our understanding of cancer origins in this area and enhance future health statistics.

## Author Contributions

Ken‐ichi Mukaisho and Sachiko Kaida authored most of the paper, whereas the other co‐authors contributed to the discussion and provided valuable insights.

## Funding

The authors have nothing to report.

## Conflicts of Interest

The authors declare no conflicts of interest.
